# On the run – the outsider and artist Ota Prouza

**DOI:** 10.1017/S2045796021000457

**Published:** 2021-09-08

**Authors:** Klaus Mecherlein

**Affiliations:** Euward Archive, Augustinum Foundation, Munich, Germany

Ota Prouza lives on the outskirts of the Czech town of Rumburg − in northern Bohemia, close to the German border. The director of *the Brtníky sheltered home* Ivana Trojanova (Mecherlein, 2018) recently furnished a small own flat here for the 58-year old. Thus, together with a fellow resident, Ota Prouza was now, for the first time in his life, given the self-determination that he always had to fight for. In the past he wandered along unfamiliar paths through the extensively wooded natural landscape. Today, he spends his free afternoons searching for pictures. Either at his desk, drawing on long paper banners, or at the municipal waste paper collection points, where, in the containers, he finds the supply of pictures for his art. Aerial views of *highways* taken by the ADAC, or photos looking down into the anonymous canyons between big-city houses. He cuts them out of newspapers and magazines and glues them into a kind of diary. The city of Rumburg and its charms, in the opinion of Ms. Trojanova, were the reasons why the unknown artist, the blank sheet, Ota Prouza flourished. This solitary wandering around and searching, for hours, is something he still does, however he now also has a home.

Ota Prouza was born in August, 1959. Not much more is known about his early childhood. At the age of eight, by an unknown route, he entered the care of an institution that, today, has established an asylum for mentally and cognitively impaired patients in a large classical style villa, previously the offices of a factory in the centre of rural Brtniky (Czech Republic). The *Domov pro osoby se zdravotním postižením der příspěvková organizace* − is, literally and in the true sense, a home for people with disabilities.

Nothing much tangible remains from Prouza's early years, his past: a photo of his mother, the name of his stepfather and a 20-year-old brother who has never been traced. Ota Prouza thus lives with the mute shadows of his past. He is skilled at dealing with them: in the middle of an ordinary conversation, he will continually invoke these family spectres. Reinventing them. Ota Prouza's often erratic communication is enveloped in a sort of uncertain knowledge - and an inquisitive researching continually underlies his piercing gaze (Fig. 1). With all the close attention he pays to the other person he always appears rapt.

**Fig. 1.**
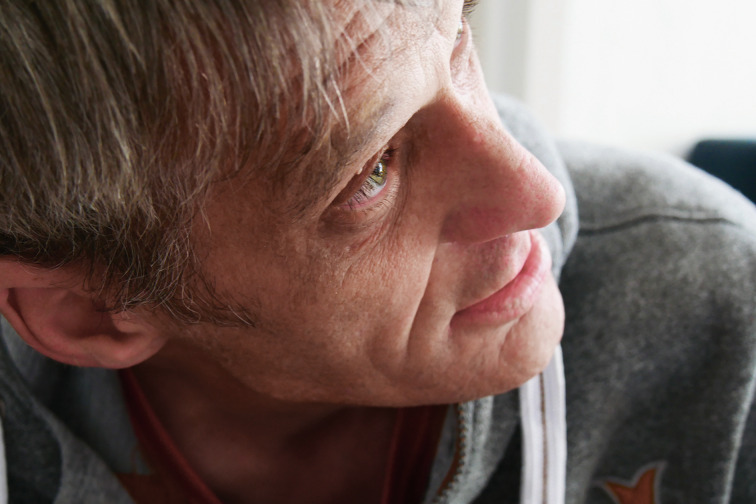
Portrait Ota Prouza, credit Klaus Mecherlein, 2018.

When Ota Prouza began drawing, and what may have been the determining factor are impossible to say now, Ota himself doesn't know. All of them, Ota's pictures, start with a long strip of pages glued together. As he is drawing, which starts more or less at all points simultaneously, Prouza continually pushes and pulls his paper strip over the rear, then the front, edge of the table, down to the floor. All that is done fairly hastily. Urgently, as though on the run.

Under the gaze of the eyes fleetingly scrutinising them, the metre-long lanes of multi-lane roads and open architectural canyons flow and fall down. Beneath the table − beyond it − then up again. This fluent, high-speed handling of the pictures is slightly reminiscent of the perforated and punched music rolls used in mechanical musical instruments of earlier times. For me, it is as though the artist wants to set the awkward scenery of his imagination elementally into motion. And, indeed, this is the effect you get when you look at the works. A vertical movement from top to bottom and from bottom to top. And at the same time, faced with this dynamism, each individual motif loses its weight. Flowing pictures. An iconic rotation. Things continually coming up close and then distancing themselves again, indeed, the distancing of the act of seeing itself.

**Fig. 2.**
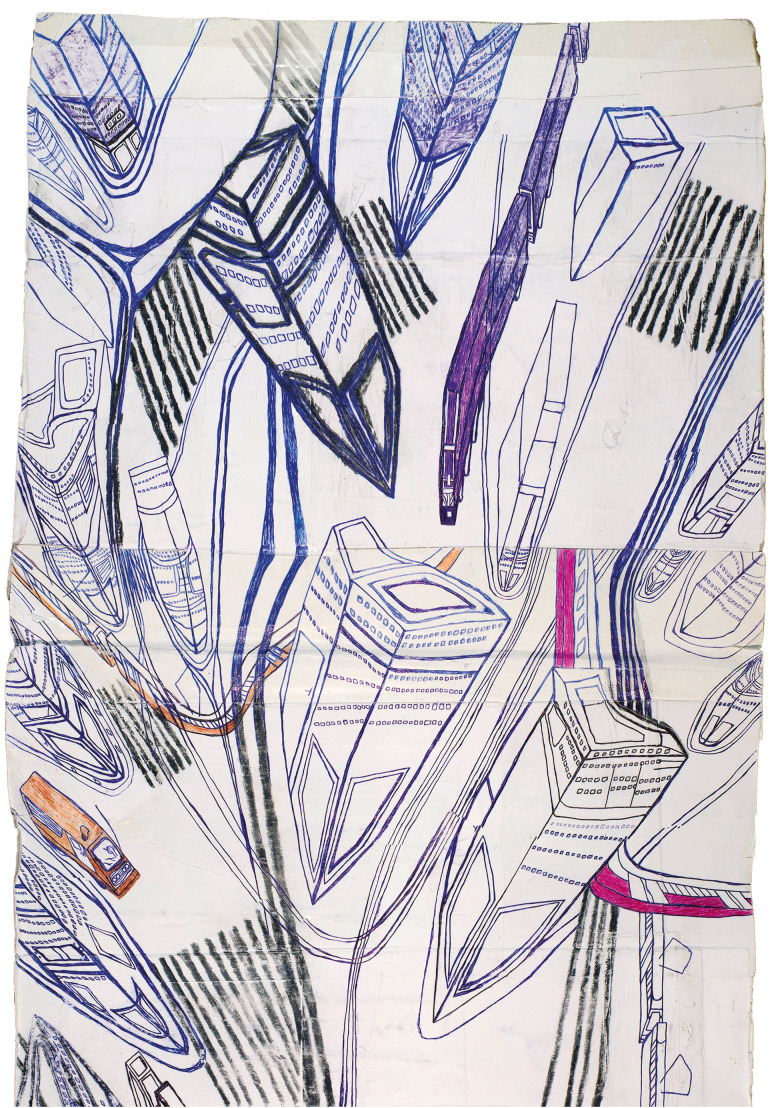
Ota Prouza Untitled (c38) 2000–2015 Detail 543 × 42 cm.

Movement! That appears to be the essence of these pages and picture strips. Picture ribbons and picture loops. Picture sheets. A transition: the pictures themselves begin to travel. If you then view them, in their full eight-meter length on the museum wall, the view down onto the world reminds you of an overflight seen through eagle's eyes. In this view up and down, along the disrupted traffic-axes of big cities one can discover great freedom and authority. The city is dissected. Its parts lie before us, spread out and clearly sorted. The haste of the traffic streams, the soot of goods vehicles, the squeaking of tram points, are inverted − into a soundlessness. The hasty restlessness of the draftsmanship, which we note in the powerful, almost beating − and always confident strokes − is inverted into silence. The traffic is regulated by a great mastery and controlling will. A conducting such as one might recognise in the operator of a model railway landscape. Or the leader of a major orchestra, whose sound and rhythm obey him at all times.

## About the author

**Klaus Mecherlein** M.A., director of the Euward-Archive of Augustinum Foundation Munich, is an art historian, exhibition curator, author and university lecturer. Following his studies in art education, art history and psychology at Ludwigs-Maximilian-University in Munich, he founded and headed the Atelier Augustinum in 1992, a studio for outsider artists in Munich, and in 2000 became the curator of Euward, the European art award by the Augustinum Stiftung. In 2001, he became director of the Eucrea Art assistant program and since 2011 he has been a part-time lecturer at Munich University of Applied Sciences. From 2009 to 2019, he has been the chairman of Eucrea e.V. He is author and co-author of various publications on Outsider Art as well as contemporary art.

Carole Tansella, Section Editor
